# Healthcare resource utilization in advanced non-small-cell lung cancer: post hoc analysis of the randomized phase 3 REVEL study

**DOI:** 10.1007/s00520-020-05459-0

**Published:** 2020-04-21

**Authors:** Edward B. Garon, Katherine B. Winfree, Cliff Molife, Zhanglin Lin Cui, Edurne Arriola, Benjamin Levy, Tarek Mekhail, Maurice Pérol

**Affiliations:** 1 David Geffen School of Medicine at UCLA/TRIO-US Network, Santa Monica, CA USA; 2 Eli Lilly and Company, Indianapolis, IN USA; 3 Hospital Del Mar, Barcelona, Spain; 4 Johns Hopkins University, Baltimore, MD USA; 5 Florida Hospital Cancer Institute, Orlando, FL USA; 6 Léon Bérard Cancer Centre, Lyon, France

**Keywords:** Non-small-cell lung cancer, Ramucirumab, Docetaxel, Healthcare resource utilization, Aggressive disease

## Abstract

**Purpose:**

In REVEL, patients with advanced non-small-cell lung cancer (aNSCLC) and patients with increased tumor aggressiveness (rapid disease progression (RDP), platinum-refractory disease (PRD), and high symptom burden (HSB)) benefited from second-line treatment with ramucirumab plus docetaxel over placebo plus docetaxel. This post hoc analysis describes healthcare resource utilization (HCRU) associated with the treatment.

**Methods:**

aNSCLC patients who had progressed during or after first-line platinum-based chemotherapy were randomized to receive docetaxel and either ramucirumab or placebo until disease progression, unacceptable toxicity, withdrawal, or death. HCRU included hospitalizations, transfusions, and concomitant medications. Categorical variables (counts and percentages) were compared using Fisher’s exact test. Continuous variables (mean, standard deviation (SD), median, minimum, and maximum) were compared using the Wilcoxon rank sum test.

**Results:**

Patient characteristics were largely similar between treatment arms. Within the intent-to-treat (ITT) population (*n* = 1253), the mean treatment duration was 19.7 and 16.9 weeks in the ramucirumab and control arms, respectively; 51.0% versus 54.9% of patients received subsequent anticancer therapy, respectively. Hospitalization rates were 41.9% versus 42.6% (*p* = 0.863), mean length of hospital stay was 14.5 days versus 11.3 days (*p* = 0.066), transfusion rates were 9.9% versus 12.3% (*p* = 0.206), and use of granulocyte colony-stimulating factors was 41.8% versus 36.6% (*p* = 0.063), respectively. No significant difference was observed in HCRU between treatment arms in both ITT population and in aggressive disease subgroups including RDP (*n* = 209), PRD (*n* = 360), and HSB (*n* = 497).

**Conclusion:**

In REVEL, the addition of ramucirumab to docetaxel did not increase HCRU among patients with aggressive aNSCLC disease. These results may help inform economic evaluation of treatment for patients with aNSCLC.

## Introduction

Lung cancer is the most common cause of cancer related death in the world, with approximately 2.09 million new cases and 1.76 million deaths predicted in 2018 [1]. Approximately 80 to 85% of all patients with lung cancer are diagnosed with non-small-cell lung cancer (NSCLC), and most patients with NSCLC present with advanced disease [2], with a 5-year survival rate of about 1% for patients with stage IV NSCLC [2, 3]. First-line platinum-based therapy, including platinum-based chemoimmunotherapy combinations [4, 5], is considered the standard of care for the majority of patients who have advanced NSCLC (aNSCLC) without actionable mutations [6–8]. Also, pembrolizumab is approved by FDA for first-line treatment of patients with high PD-L1 expression [9, 10].

Current guidelines for the broader aNSCLC population who fail first-line therapy recommend second-line treatment with immune checkpoint inhibitors (if not received with first-line therapy), single-agent chemotherapy, or an antiangiogenic agent (i.e., ramucirumab) plus docetaxel [7, 8]. As the use of first-line treatments that include immunotherapies increases, the use of immunotherapies as second-line treatment options may decrease in the absence of clinical data on sequential immunotherapy. Ramucirumab, an antiangiogenic vascular endothelial growth factor receptor-2 inhibitor, is approved by the Food and Drug Administration (FDA) and European Medicines Agency (EMA) for use in combination with docetaxel for the treatment of patients with metastatic NSCLC with disease progression on or after platinum-based chemotherapy. This approval was based on the phase 3 REVEL study, which demonstrated improved survival with the addition of ramucirumab to docetaxel in second-line treatment [11]. Overall, adverse events (AEs) were manageable with dose adjustments and supportive care, and the addition of ramucirumab did not negatively affect patients’ quality of life [12]. These findings were directionally consistent across the majority of subgroups evaluated in the trial, including patients with aggressive disease features, including rapid (≤ 12 weeks) disease progression or refractory disease (best response of progressive disease) during the platinum regimen [13–15]. Patients with aggressive disease on initial platinum-based therapy, including those with high symptom burden, represent a vulnerable subgroup with poorer prognosis and suboptimal therapy options in the post-progression setting [11].

The economic burden of lung cancer faced by healthcare systems is substantial, and healthcare spending continues to rise. Understanding the economic implications of a given treatment is important to optimize care; payers and providers may consider resource use along with the clinical efficacy and toxicity of a given treatment to support decision-making. In REVEL, no healthcare cost information was collected, and within-trial cost analysis was not specified or conducted. Likewise, the present analysis did not apply unit costs to healthcare resource utilization (HCRU) measures, as absolute values of healthcare costs are often not generalizable to individual health systems or institutions due to high regional variation in costs both within and outside the USA [16, 17]. Nonetheless, HCRU data from this analysis may provide a key input parameter for institution-specific economic evaluations comparing the costs and clinical effects of adding ramucirumab to docetaxel for second-line treatment of aNSCLC.

This study used data from REVEL to assess the HCRU associated with ramucirumab plus docetaxel compared with placebo plus docetaxel. While Garon et al. (2014) reported the frequency of hospital admissions, transfusions, and usage of growth factors in the REVEL primary ITT population, further details on these and additional HCRU data for the ITT population along with comprehensive HCRU for aggressive disease subgroups are presented in this paper.

## Methodology

### Study design, patients, and treatment

The design and key clinical results of the REVEL study as well as subgroup analyses of patients with advanced NSCLC have been previously published (NCT01168973) [11, 13–15]. Briefly, REVEL was an international (216 investigative sites in 26 countries), multicenter, randomized double blind trial of patients with squamous or non-squamous NSCLC who had progressed during or after first-line platinum-based chemotherapy. Patients were randomized to receive docetaxel 75 mg/m^2^ and either ramucirumab (10 mg/kg) or placebo on day 1 of a 21-day cycle until disease progression, unacceptable toxicity, withdrawal, or death. The primary endpoint was the overall survival, and secondary endpoints included the safety and toxicity profile for study treatment, including the associated HCRU [11].

This analysis examined HCRU among the ITT population and subgroups of patients with aggressive aNSCLC from the REVEL study. Aggressive disease was defined as having either rapid disease progression (RDP) on first-line treatment, platinum-refractory disease (PRD), or high symptom burden (HSB) at baseline. Patients with RDP had time to progression of ≤ 12 weeks on first-line therapy. Patients with PRD had progressive disease as their best response to first-line therapy. Patients with HSB had an average symptom burden index score greater than the median at baseline, determined using the patient-reported Lung Cancer Symptom Scale. The three subgroups with aggressive disease were not mutually exclusive, and patients frequently fell into multiple subgroups.

### Healthcare resource utilization

Healthcare resource utilization measures were collected during the study treatment period or within 30 days after study treatment discontinuation and included treatment duration, transfusions, hospitalizations, length of hospital stay, palliative and supportive care (e.g., concomitant medications) for other disease-related symptoms or treatment-related toxicity, and post-discontinuation therapy. Treatment duration was defined as the time between the first and the last infusion of the study drug. Transfusions were characterized by transfused blood product (e.g., packed red blood cells, platelets, or whole blood). For hospitalizations that were still ongoing at the time of analysis, the hospital discharge date was imputed with the last contact date. Relevant concomitant medications were identified based on anticipated supportive care for disease-related symptoms and for toxicity associated with treatment in the REVEL study. The use of post-discontinuation therapy was not specified per protocol, and following study therapy discontinuation, patients could receive additional anticancer therapies at the discretion of the investigator.

### Statistical analyses

Categorical variables were summarized by treatment arms as counts and percentages and compared using the Fisher’s exact test. Continuous variables were summarized by treatment arms as mean, standard deviation (SD), median, minimum, and maximum and compared using the Wilcoxon rank sum test. All statistical analyses were descriptive, without baseline risk adjustment, and performed separately within each subgroup using SAS, version 9.3, software (SAS Institute Inc., Cary, NC).

## Results

### Patient characteristics

For the REVEL study, 1825 patients were screened, of whom 1253 patients were randomly allocated to treatment. Overall, baseline patient characteristics and prior therapies were largely balanced between treatment arms (Table 1). Patients were also analyzed for the presence of aggressive disease. Of 1253 randomized patients in the REVEL ITT population, 360 (28.7%) had PRD, 209 (16.7%) had RDP on first-line treatment, and 497 (39.7%) had HSB at baseline. There was a considerable overlap between aggressive disease subgroups, particularly between patients with PRD and RDP. Specifically, over 80% of patients with RDP met the criteria for PRD, while approximately one third (34%) of the patients with HSB were also refractory to platinum therapy.

**Table 1:** Baseline patient characteristics

Patient demographic characteristics	ITT population		Refractory patients		Rapidly progressing patients (≤ 12 weeks)		High symptom burden patients	
	Ram + Doc % (*n* = 628)	Pbo + Doc % (*n* = 625)	Ram + Doc % (*n* = 178)	Pbo + Doc % (*n* = 182)	Ram + Doc % (*n* = 111)	Pbo + Doc % (*n* = 98)	Ram + Doc % (*n* = 228)	Pbo + Doc % (*n* = 269)
Gender (male/female)	67/33	66/34	77/23	70/30	73/27	70/30	69/31	68/32
Age (median/% of patients with age ≥ 65 years)	62 years/38	61 years/35	63 years/35	60 years/24	62 years/29	59 years/24	63 years/40	61 years/30
Race, White Asian Black/African American	84 12 3	80 14 3	84 13 2	82 16 1	87 8 3	82 17 0	84 11 3	84 13 3
Region. East Asia/rest of the world	7/93	7/93	–	–	5/95	9/91	4/96	6/94
ECOG PS (0/1)	33/67	32/68	30/70	27/73	33/67	21/79	21/79	24/76
Prior maintenance (yes/no)	21/79	23/77	4/96	7/93	1/99	3/97	22/78	23/77
Histology (non-squamous/ squamous)	74/25	72/27	73/26	71/ 27	76/23	77/24	76/24	72/27
Smoking status (ever/never)	82/17	77/23	85/15	78/21	87/13	80/19	85/15	77/23
Prior bevacizumab (yes/no)	14/86	15/85	11/89	8/92	14/86	9/91	16/84	16/84
Prior taxane (yes/no)	24/76	24/76	22/78	19/81	20/80	16/84	27/73	23/78
Best response to prior chemo CR, PR or SD/PD	67 28	67 29	0 100	0 100	16 82	12 85	63 32	64 33

*CR* complete response; *Doc* docetaxel; *ECOG* Eastern Cooperative Oncology Group; *ITT* intent to treat; *Pbo* placebo; *PD* progressive disease; *PR* partial response; *PS* performance status; *Ram* ramucirumab; *SD* stable disease

The median age for the ITT population, RDP, PRD, and HSB subgroups ranged from 60 to 63 years, and the proportion of patients who were 65 years and older was respectively 38%, 29%, 35%, and 38% for the ramucirumab arm and 35%, 24%, 24%, and 30% for the control arm. While the proportion of males was consistent in the ramucirumab and control arms for the ITT population, it tended to be higher for the ramucirumab arm, relative to the control arm, in the RDP and PRD subgroups. Overall, at least two thirds of patients had an Eastern Cooperative Oncology Group performance status (ECOG PS) of 1, and as expected, the proportion of patients with an ECOG PS of 1 was numerically higher for the HSB subgroup. In general, ECOG PS was balanced between treatment arms, although it appeared to be higher in the ramucirumab arm for patients with RDP. Overall, the percentage of prior smokers seemed to be slightly higher in the ramucirumab arm than the control arm. Prior treatment with bevacizumab or taxane-based therapy was balanced in the overall ITT population, but it trended higher in the ramucirumab arm for aggressive disease subgroups. On the other hand, prior maintenance treatment was more common in the ITT population when considered alongside the aggressive disease subgroups.

### Resource utilization

#### Duration of study therapy

For the REVEL ITT population, the mean (SD) duration of therapy was 19.7 (16.9) weeks for the ramucirumab arm and 16.9 (16.0) weeks for the control arm. Similarly, in patients with RDP, the mean (SD) duration of therapy was 17.0 (15.3) weeks for the ramucirumab arm and 14.0 (16.1) weeks for the control arm (*p* = 0.0447), with a mean (SD) of 5.5 (4.9) and 4.5 (5.1) infusions received (*p* = 0.0134), respectively. These data were directionally similar for PRD and HSB subgroups and for the ITT population. However, treatment duration appeared lower overall for subgroups with aggressive disease relative to the ITT population, especially in the control arm (Table 2).

**Table 2:** Number and duration of infusions by subgroup and treatment arm

Cycles of infusions	ITT population		Refractory patients		Rapidly progressing patients (≤ 12 weeks)		High symptom burden patients	
	Ram + Doc (*n* = 627)	Pbo + Doc (*n* = 618)	Ram + Doc (n = 178)	Pbo + Doc (*n* = 180)	Ram + Doc (*n* = 109)	Pbo + Doc (*n* = 97)	Ram + Doc (*n* = 229)	Pbo + Doc (*n* = 264)
Number of infusions of therapy received per patient, mean (SD), median (range)	6.2 (5.38) 5.0 (1–38)	5.4 (5.09) 4.0 (1–42)	5.4 (5.20) 4.0 (1–23)	4.6 (4.56) 3.0 (1–32)	5.5 (4.89) 4.0 (1–23)*	4.5 (5.12) 2.0 (1–32)	5.2 (4.57 4.0 (1–25)*	4.5 4.21) 3.0 (1–21)
Duration of treatment in weeks per patient, mean (SD), median (range)	19.7 (16.90) 15.0(3–118)	16.9 (15.97) 12.0(3–133)	17.2 (16.44) 12.0 (3–76)	14.3 (14.35 9.0 (3–103)	17.0 (15.31) 12.0 (3–69)*	14.0 (16.07) 7.1 (3–103)	16.6 (14.42) 12.0 (3–84)*	14.0 (13.30) 9.0 (3–71)

*Doc* docetaxel; *ITT* intent to treat; *Pbo* placebo; *Ram* ramucirumab; *SD* standard deviation

*Only significant treatment differences (*p* values < 0.05) are indicated with asterisks. Comparisons were based on Wilcoxon rank sum test.

#### Hospitalizations

The hospitalization rate for the ITT population was 41.9% and 42.6% in the ramucirumab and control arm, respectively. For patients with RDP, the hospitalization rate was 41.3% in the ramucirumab arm and 48.5% in the control arm (*p* = 0.328) (Table 3). The mean (SD) length of hospital stay per patient was observed to be 14.5 (16.5) days in the ramucirumab arm and 11.3 (9.9) days in control arm in the ITT population. For the RDP population, the mean (SD) length of hospital stay per patient was 11.3 (10.7) days in the ramucirumab arm and 13.1 (12.8) days in the control arm (*p* = 0.685). The average (SD) number of hospitalizations per patient was 1.6 (1.0) in the ramucirumab arm and 1.6 (1.1) in the control arm (*p* = 0.627). These data were directionally consistent among the RDP, PRD, and HSB subgroups, as well as the overall ITT population, although hospital length of stay in the ramucirumab group was numerically lower for the RDP group. Compared with the ITT population, hospitalization rates tended to be numerically higher in the control arm in patients with aggressive disease, especially in the HSB group (Table 3).

**Table 3:** Number and duration of hospitalization by subgroup and treatment arm

Hospitalization	ITT population		Refractory patients		Rapidly progressing patients (≤ 12 weeks)		High symptom burden patients	
	Ram + Doc (*n* = 627)	Pbo + Doc (*n* = 618)	Ram + Doc (*n* = 178)	Pbo + Doc (*n* = 180)	Ram + Doc (*n* = 109)	Pbo + Doc (*n* = 97)	Ram + Doc (*n* = 229)	Pbo + Doc (*n* = 264)
Patients with ≥ 1 hospitalizatio*n*; *n* (%)	263 (41.9)	263 (42.6)	76 (42.7)	81 (45.0)	45 (41.3)	47 (48.5)	111 (48.5)	133 (50.4)
Hospitalizations (days), mean (SD), median (range)	14.5 (16.5) 9.0 (1–128)	11.3 (9.9) 8.0 (1–56)	15.2 (21.1) 9.0 (1–128)	11.7 (10.2) 8.0 (1–56)	11.3 (10.7) 8.0 (1–56)	13.1 (12.8) 9.0 (2–56)	15.3 (16.3) 10.0 (1–128)	13.2 (11.5) 10.0 (1–56)
Hospitalizations (admissions), mean (SD), median (range)	1.6 (0.9) 1.0 (1–7)	1.5 (0.9) 1.0 (1–8)	1.5 (1.0) 1.0 (1–6)	1.5 (1.0) 1.0 (1–8)	1.6 (1.0) 1.0 (1–6)	1.6 (1.1) 1.0 (1–8)	1.7 (1.1) 1.0 (1–7)	1.5 (0.8) 1.0 (1–6)

*Doc* docetaxel; *ITT* intent to treat; *Pbo* placebo; *Ram* ramucirumab; *SD* standard deviation

The mean of hospitalizations is calculated as the total number of hospitalizations divided by number of patients with ≥1 hospitalization.

Comparisons of patients with hospitalizations were based on Fisher’s exact test; comparisons of average days and admissions were based on Wilcoxon rank sum test. None of the treatment comparisons were statistically significant at *p* value = 0.05 level.

#### Transfusions and supportive care

In the ITT population, the transfusion rate was 9.9% in the ramucirumab arm and 12.3% in the control arm. For patients with RDP, the transfusion rate was 10.1% in the ramucirumab arm and 16.5% for the control arm (*p* = 0.216) (Table 4). This finding was directionally similar for the HSB group, while transfusion rates were numerically similar between treatment arms in patients with PRD. Packed red blood cells were used most often for blood transfusions (Table 4).

**Table 4:** Number and type of transfusion by subgroup and treatment arm

Transfusio*n*; *n* (%)	ITT population		Refractory patients		Rapidly progressing patients (≤ 12 weeks)		High symptom burden patients	
	Ram + Doc (*n* = 627)	Pbo + Doc (*n* = 618)	Ram + Doc (*n* = 178)	Pbo + Doc (*n* = 180)	Ram + Doc (*n* = 109)	Pbo + Doc (*n* = 97)	Ram + Doc (*n* = 229)	Pbo + Doc (*n* = 264)
According to number of transfusion/s								
Patients with no transfusion	–	–	154 (86.5)	155 (86.1)	98 (89.9)	81 (83.5)	203 (88.6)	224 (84.8)
Patients with ≥ 1 transfusion	62 (9.9)	76 (12.3)	24 (13.5)	25 (13.9)	11 (10.1)	16 (16.5)	26 (11.4)	40 (15.2)
Transfusion type								
Blood cells, packed human	5 (0.8)	9 (1.5)	1 (0.6)	3 (1.7)	1 (0.9)	1 (1.0)	0*	6 (2.3)
Packed red blood cells	53 (8.5)	72 (11.7)	21 (11.8)	25 (13.9)	10 (9.2)	15 (15.5)	22 (9.6)	38 (14.4)
Platelets	4 (0.6)	1 (0.2)	1 (0.6)	0	-	-	1 (0.4)	0
Whole blood	4 (0.6)	2 (0.3)	1 (0.6)	1 (0.6)	0	2 (2.1)	3 (1.3)	0

*Doc* docetaxel; *ITT* intent to treat; *Pbo* placebo; *Ram* ramucirumab

*Only significant treatment differences (*p* values < 0.05) are indicated with asterisks. Comparisons between treatment arms were based on Fisher’s exact test.

While the majority of patients in REVEL received at least one concomitant medication overall and rates of use of concomitant medication were statistically similar between treatment arms, numerically higher rates were observed in aggressive disease subgroups (> 93%) relative to the ITT population (about 88%). Approximately 79.4% of ITT patients in the ramucirumab arm and 78.3% patients in the control arm received relevant concomitant medications (*p* = 0.677), as defined in the categories in Table 5. The proportion of patients receiving relevant concomitant medications was 72.5% in the ramucirumab arm and 78.4% in the control arm for patients with RDP (*p* = 0.338) and 70.8% and 71.1%, respectively, in the PRD subgroup (*p* = 1.000). These rates were generally lower than those for the HSB subgroup (79.5% vs 78.4%, *p* = 0.825) (Table 5).

**Table 5:** Number and type of concomitant mediation use by subgroup and treatment arm

Concomitant medications; *n* (%)	ITT population		Refractory patients		Rapidly progressing patients (≤ 12 weeks)		**High symptom burden patients**	
	Ram + Doc (*n* = 627)	Pbo + Doc (*n* = 618)	Ram + Doc (*n* = 178)	Pbo + Doc (*n* = 180)	Ram + Doc (*n* = 109)	**Pbo + Doc (*n*** **= 97)**	**Ram + Doc (*n*** **= 229)**	**Pbo + Doc (*n*** **= 264)**
Amount of concomitant medication								
Patients receiving no concomitant medication	–	–	5 (2.8)	12 (6.7)	3 (2.8)	4 (4.1)	4 (1.7)	12 (4.5)
Patients receiving ≥ 1 concomitant medication	554 (88.4)	537 (86.9)	173 (97.2)	168 (93.3)	106 (97.2)	93 (95.9)	225 (98.3)	252 (95.5)
Patients with any concomitant medication from the relevant list	498 (79.4)	484 (78.3)	126 (70.8)	128 (71.1)	79 (72.5)	76 (78.4)	182 (79.5)	207 (78.4)
Type of concomitant medication								
Anticoagulants/antithrombotics	72 (11.5)	82 (13.3)	19 (10.7)	23 (12.8)	13 (11.9)	14 (14.4)	32 (14.0)	38 (14.4)
Erythropoietic agents	19 (3.0)	23 (3.7)	5 (2.8)	5 (2.8)	3 (2.8)	4 (4.1)	9 (3.9)	11 (4.2)
Antihypertensive	159 (25.4)*	109 (17.6)	26 (14.6)	30 (16.7)	16 (14.7)	19 (19.6)	63 (27.5)*	47 (17.8)
Antiemetic	129 (20.6)	115 (18.6)	30 (16.9)	30 (16.7)	20 (18.3)	18 (18.6)	50 (21.8)	51 (19.3)
Antibiotics	247 (39.4)*	197 (31.9)	54 (30.3)	53 (29.4)	29 (26.6)	33 (34.0)	94 (41.0)	88 (33.3)
Antivirals	6 (1.0)	6 (1.0)	1 (0.6)	0	–	–	1 (0.4)	1 (0.4)
Antifungals	90 (14.4)	51 (8.3)	23 (12.9)	12 (6.7)	14 (12.8)	8 (8.2)	24 (10.5)	25 (9.5)
GCSF/GM-CSF	262 (41.8)	226 (36.6)	60 (33.7)	65 (36.1)	39 (35.8)	35 (36.1)	87 (38.0)	93 (35.2)
Analgesics	228 (45.9)	290 (46.9)	78 (43.8)	73 (40.6)	46 (42.2)	44 (45.4)	116 (50.7)	128 (48.5)

*Doc* docetaxel; *GCSF/GM-CSF* granulocyte colony-stimulating factors and granulocyte macrophage colony-stimulating factors; *ITT* intent to treat; *Pbo* placebo; *Ram* ramucirumab

*Only significant treatment difference (*p* values < 0.05) are indicated with asterisks. Comparisons between treatment arms were based on Fisher’s exact test.

As expected, analgesics were the most commonly used concomitant medications among both the ITT population (> 45%) and aggressive disease subgroups (> 40%) [18] (Table 5). The use of granulocyte colony-stimulating factors and granulocyte-macrophage colony-stimulating factors (G-CSFs/GM-CSFs) tended to be higher in the ramucirumab arm among the ITT population (41.8% vs. 36.6%, *p* = 0.063), while usage rates were similar between treatment arms for aggressive disease subgroups. Antihypertensive medications were used more frequently overall and in the ramucirumab arm among the ITT population (25.4 vs. 17.6, *p* < 0.001) and the HSB subgroup (27.5% vs. 17.8%, *p* = 0.012). The use of antihypertensive medications was numerically lower in the ramucirumab arm than in the control arm for RDP (14.7% vs 19.6%, *p* = 0.360) and PRD (14.6% vs 16.7%, *p* = 0.663) subgroups. The use of antibiotics followed a similar pattern. Specifically, antibiotic usage rates were 39.4% in the ramucirumab arm and 31.9% in the control arm among the overall ITT population (*p* = 0.006), 26.6% and 34.0% in patients with RDP (*p* = 0.288), 30.3% and 29.4% in patients with PRD (*p* = 0.908), and 41.0% and 33.3% in patients with HSB (*p* = 0.092). Antifungals tended to be used more frequently in the ramucirumab arm among the ITT population (*p* < 0.001) and aggressive disease subgroups (*p* > 0.05). There was no difference in the use of antivirals or antiemetics between treatment arms across the ITT population or aggressive disease subgroups (Table 5).

#### Post-discontinuation therapy

For the ITT population, approximately 53% of patients received post-discontinuation anticancer therapy, most of which was in the form of systemic therapy (> 45%), followed by radiotherapy (> 15%), and then surgery (~ 1%) (Table 6), consistent with recommendations from clinical guidelines [6, 7]. The number of patients receiving post-discontinuation therapy was higher in the control arm in comparison with the treatment arm, although this difference was not statistically significant. As observed in the overall ITT population, there was no significant difference between treatment arms in the proportion of patients receiving post-discontinuation therapy among aggressive disease subgroups (*p* > 0.05). However, rates of post-discontinuation therapy tended to be higher in the ramucirumab arm than in the control arm in RDP (55.1% vs 51.1%) and PRD (48.3% vs 45.7%) subgroups, whereas usage rates tended to be lower in the ramucirumab arm in the HSB subgroup (46.4% vs 49.8%) and the ITT population (51.0% vs 54.9%). Overall, epidermal growth factor receptor tyrosine kinase inhibitors (EGFR-TKIs) were the most commonly used systematic drug treatment (Table 6).

**Table 6:** Number and type of post-discontinuation therapy received by patients according to subgroup and treatment arm

Post-discontinuation therapy; *n* (%)	ITT population		Refractory patients		Rapidly progressing patients (≤ 12 weeks)		High symptom burden patients	
	Ram + Doc (*n* = 628)	Pbo + Doc (*n* = 625)	Ram + Doc (*n* = 149)	Pbo + Doc (*n* = 162)	Ram + Doc (*n* = 98)	Pbo + Doc (*n* = 90)	Ram + Doc (*n* = 196)	Pbo + Doc (*n* = 239)
Patients with at least 1 post-discontinuation anticancer therapy	320 (51.0)	343 (54.9)	72 (48.3)	74 (45.7)	54 (55.1)	46 (51.1)	91 (46.4)	119 (49.8)
Type of therapy								
Surgery	8 (1.3)	11 (1.8)	1 (0.7)	0	2 (2.0)	1 (1.1)	2 (1.0)	4 (1.7)
Radiotherapy	99 (15.8)	103 (16.5)	25 (16.8)	26 (16.0)	15 (15.3)	15 (16.7)	34 (17.3)	32 (13.4)
Systemic therapy	285 (45.4)	302 (48.3)	65 (43.6)	65 (40.1)	50 (51.0)	41 (45.6)	79 (40.3)	105 (43.9)
Systemic therapy drug								
EGFR-TKI	118 (18.8)	133 (21.3)	25 (16.8)	31 (19.1)	21 (21.4)	19 (21.1)	29 (14.8)	43 (18.0)
Gemcitabine	77 (12.3)	73 (11.7)	14 (9.4)	14 (8.6)	13 (13.3)	9 (10.0)	20 (10.2)	26 (10.9)
Pemetrexed	66 (10.5)	46 (7.4)	19 (12.8)	11 (6.8)	11 (11.2)	5 (5.6)	19 (9.7)	13 (5.4)
Vinorelbine	59 (9.4)	64 (10.2)	13 (8.7)	13 (8.0)	12 (12.2)	7 (7.8)	19 (9.7)	22 (9.2)
Platinum	58 (9.2)	49 (7.8)	8 (5.4)	7 (4.3)	9 (9.2)	4 (4.4)	22 (11.2)	18 (7.5)
Taxane	38 (6.1)	45 (7.2)	8 (5.4)	7 (4.3)	7 (7.1)	4 (4.4)	7 (3.6)	13 (5.4)
Bevacizumab	12 (1.9)	8 (1.3)	1 (0.7)	1 (0.6)	1 (1.0)	1 (1.1)	2 (1.0)	2 (0.8)
ALK Inhibitor	6 (1.0)	6 (1.0)	0	1 (0.6)	1 (1.0)	0	1 (0.5)	3 (1.3)

*ALK* anaplastic lymphoma kinase; *Doc* docetaxel; *EGFR* epidermal growth factor receptor; *TKI* tyrosine kinase inhibitor; *ITT* intent to treat; *Pbo* placebo; *Ram* ramucirumab;

None of the treatment comparisons were statistically significant at *p* value = 0.05 level.

## Discussion

This exploratory analysis characterized HCRU for the ITT population as well as subgroups of patients in the REVEL study. In the ITT population, the frequency and length of hospitalization, transfusion rates, and use of concomitant and post-discontinuation therapy were largely similar between the ramucirumab and control arms, and as expected, the duration of study treatment was longer for the ramucirumab arm. These findings were consistent with those observed among RDP, PRD, or HSB subgroups. Considered together, the results suggest that the survival benefit and manageable safety profile provided by the addition of ramucirumab to docetaxel as second-line treatment of patients with aNSCLC were not at the expense of increased HCRU, irrespective of aggressiveness of disease or the longer time on treatment over which resource use data were collected relative to the control arm. As HCRU is a major cost driver for patients who have lung cancer, these data may be relevant for clinicians and value-based decision-makers who need to make rational and informed decisions about patient care and allocation of resources [19–21].

The longer treatment duration in the ramucirumab arm reflects the delay in disease progression with ramucirumab plus docetaxel therapy in REVEL [11]. The duration of progression-free survival (PFS) benefit with ramucirumab plus docetaxel was 1.5 months in the overall ITT population (4.5 vs 3.0 months; hazard ratio (HR) 0.76; 95% confidence interval (CI) 0.68–0.86) [11]. For patients with RDP on prior platinum-based therapy, the duration of benefit in PFS with ramucirumab plus docetaxel was 2.0 months (3.6 vs 1.6 months; HR 0.73; 95% CI 0.55–0.97) [15]. Similarly, for PRD and HSB, the duration of benefit in PFS was 1.5 months (4.0 vs 2.5 months; HR 0.71; 95% CI 0.57–0.88) and 1.4 months (4.0 vs 2.6 months; HR 0.749; 95% CI 0.62–0.91), respectively [13, 14]. The duration of PFS benefit was similar in the ITT population and aggressive disease subgroups, suggesting that adding ramucirumab to docetaxel may be a worthwhile consideration for previously treated patients with aNSCLC.

The demonstration of no added HCRU burden from the addition of ramucirumab is in line with the acceptable safety and quality of life profiles for ramucirumab plus docetaxel relative to docetaxel alone, as observed in the REVEL primary and subgroup analyses. In REVEL, the incidence of grade ≥ 3 treatment-emergent adverse events (TEAEs), serious AEs, TEAEs leading to discontinuation, and fatal TEAEs was not significantly different between treatment arms in the ITT population and in patients with aggressive disease. This balance was further supported by secondary analyses from REVEL showing similar patient-reported symptoms and quality of life, as well as clinician-reported patient functioning, between treatment arms [12]. Overall, the most common grade ≥ 3 AEs in the ramucirumab arm were neutropenia, severe infections, febrile neutropenia, fatigue, leucopenia, and hypertension. Within the ITT population, patients were most often hospitalized for febrile neutropenia (13.1% vs 8.1%), pneumonia (5.4% vs 5.0%), and neutropenia (3.8% vs 4.0%). The lack of significant difference in the rates and length of hospitalization between treatment arms, particularly those related to the management of neutropenia, is an important consideration for healthcare decision-makers, as hospitalizations are known to be a major cost driver for patients with aNSCLC in several countries [19, 21–25].

While differences in AEs were not statistically significant, the overall previously reported numerical differences in specific AEs may explain the observed numerical differences in HCRU between treatment arms in REVEL [11]. For example, transfusion rates for the overall ITT population trended lower in the ramucirumab arm, consistent with the numerically lower rate of grade ≥ 3 anemia in the ramucirumab arm (3%) compared with the control arm (6%). Additionally, the use of G-CSF/GM-CSF (42% vs 37%), antibiotics (39% vs 32%), antifungals (14% vs 8%), and antihypertensive agents (25% vs. 18%) was numerically higher in the ramucirumab arm, in line with the higher incidence of neutropenia (49% vs 39%), febrile neutropenia (16% vs 10%), severe infections (26% vs 20%), and treatment-emergent hypertension (6% vs 2%) in that arm versus the control arm. Overall, nearly half of the patients in REVEL received subsequent systemic anticancer therapy after study treatment discontinuation. The use of post-discontinuation anticancer therapy was generally similar (ITT, 51% vs 55%) between the ramucirumab and control arms, respectively, as was use of specific anticancer treatments (e.g., EGFR-TKIs), suggesting that the observed treatment benefit observed in the REVEL ITT population and aggressive disease subgroups was due to a treatment effect of adding ramucirumab to docetaxel.

Although HCRU findings for the ITT population and patients with aggressive disease subgroups were largely consistent, there were some notable directional and numerical differences across the cohorts. For example, while hospitalization rates were directionally consistent across the overall ITT population and aggressive disease subgroups, rates were numerically higher for the control arm among aggressive disease subgroups, and the average length of hospital stay was numerically lower in the ramucirumab arm for patients with RDP. Relative to the overall ITT population and HSB subgroup, RDP and PRD subgroups appeared to have received numerically more concomitant medications overall and fewer relevant concomitant medications. The use of G-CSF/GM-CSF was similar between treatment arms in the aggressive disease subgroups, whereas the usage of G-CSF/GM-CSF was higher in the ramucirumab arm in the ITT population. The use of antihypertensive agents or antibiotics in the ramucirumab arm, compared with the control arm, was lower in RPD and PRD subgroups but trended higher in the HSB group and the ITT population. Post-discontinuation therapy in the ramucirumab arm appeared to be slightly higher than that in the control arm in RDP and PRD subgroups but slightly lower in the HSB group and the ITT population. The rationale for these varied numerical trends across populations was not evaluated in the current analysis, although it is possible that the variations were due to differences in baseline risks, prior treatments, or AE incidence rates among the ITT population and aggressive disease subgroups, or they may have been due to chance.

While this study is the first comprehensive examination of the impact of ramucirumab plus docetaxel on HCRU, some caveats should be noted when interpreting the findings. First, HCRU data were based on a randomized clinical trial with specific inclusion and exclusion criteria that may not reflect real-world clinical practice. Furthermore, summarized multinational HCRU in this study may not fully account for possible differences in treatment practices and patterns of resource use between countries or healthcare systems. For example, if a large proportion of patients come from countries for which the threshold for hospitalization differs considerably from that in other countries, the difference in HCRU between treatment arms may be under- or overestimated. Lastly, analyses were limited to direct HCRU and did not account for indirect HCRU such as lost productivity and caregiver time, which would be required for economic evaluations that adopt a societal perspective. With the similar rates of adverse events and HCRU between arms, indirect HCRU would likely not differ significantly by treatment; however, this was not studied here specifically.

## Conclusion

While patients in the REVEL study had longer treatment duration with the ramucirumab arm than the control arm, the addition of ramucirumab to docetaxel did not increase most HCRU. The HCRU among the overall ITT population was generally consistent with patients with aggressive disease. These data may aid decision-making by providing inputs for future economic evaluation of treatment for aNSCLC. However, the results must be tempered as HCRU from the clinical trial setting may underestimate that in routine clinical practice.
